# A Wireless Sensor Network for the Real-Time Remote Measurement of Aeolian Sand Transport on Sandy Beaches and Dunes

**DOI:** 10.3390/s18030820

**Published:** 2018-03-08

**Authors:** Alessandro Pozzebon, Irene Cappelli, Alessandro Mecocci, Duccio Bertoni, Giovanni Sarti, Fernanda Alquini

**Affiliations:** 1 Department of Information Engineering and Mathematical Sciences, University of Siena, 53100 Siena, Italy; irene.cappelli@student.unisi.it (I.C.); alessandro.mecocci@unisi.it (A.M.); 2 Department of Earth Sciences, University of Pisa, 56126 Pisa, Italy; duccio.bertoni@unipi.it (D.B.); giovanni.sarti@unipi.it (G.S.); 3 Department of Architecture and Urbanism, Faculty of Uniasselvi Group FAMEG, Guaramirim 89270-000, Brazil; alquini@gmail.com

**Keywords:** WSN, ZigBee, sand transport, load cell, IoT, wind dynamics, coastal dynamics, environmental monitoring

## Abstract

Direct measurements of aeolian sand transport on coastal dunes and beaches is of paramount importance to make correct decisions about coast management. As most of the existing studies are mainly based on a statistical approach, the solution presented in this paper proposes a sensing structure able to orient itself according to wind direction and directly calculate the amount of wind-transported sand by collecting it and by measuring its weight. Measurements are performed remotely without requiring human action because the structure is equipped with a ZigBee radio module, which periodically sends readings to a local gateway. Here data are processed by a microcontroller and then transferred to a remote data collection centre, through GSM technology. The ease of installation, the reduced power consumption and the low maintenance required, make the proposed solution able to work independently, limiting human intervention, for all the duration of the expected experimental campaign. In order to analyze the cause-effect relationship between the transported sand and the wind, the sensing structure is integrated with a multi-layer anemoscope-anemometer structure. The overall sensor network has been developed and tested in the laboratory, and its operation has been validated in field through a 48 h measurement campaign.

## 1. Introduction

Sediment transport is a branch of sedimentology that has been deeply analyzed and studied [1,2,3,4]. Every aspect related to sediments and grains depends on how they are entrained, distributed, and deposited. That applies for each environment, even though the processes may be so different should the setting be marine, coastal, fluvial, or glacial. In general terms, grains are entrained as a function of particle size with increasing flow speed (except for cohesive sediment, such as clay and silt, which require higher erosion velocity as cohesive forces are significant), transported according to flow direction, and finally deposited once flow speed decreases under the critical velocity for deposition for each particle size [5]. Erosion and accumulation areas are pointed out, but the mechanisms responsible to move the grains from the erosion site to the deposition site are extremely variable and complex.

As a matter of fact, intense scientific production has been provided about sediment transport from a variety of environments: glacial [6,7], fluvial [8,9,10], marine [11,12,13], and coastal [14,15,16]; it has also been addressed on another planet, Mars [17]. In particular, in a coastal area sediment transport defines how grains offset, the patterns of movement, and how much sediment is transported, which in turn are influenced by several independent factors such as wave parameters (e.g., direction, height, period, frequency), currents (e.g., longshore or cross-shore, continuous or intermittent), tides (ebb and flood), fluvial discharge to the sea (e.g., suspended sediment concentration, bedload rate). Sediment transport rates are crucial in the coastal environment, because the evolution of a littoral area hinges on the equilibrium—or lack of it—between sediments feeding the system and sediments leaving the system.

Even though coastal erosion is also induced by natural factors (such as eustatism, sea-level fluctuations, subsidence), man has had a great impact in the sense that contributed to accentuate processes that usually showed their effects in the longer term rather than in a century. As a matter of fact, coastal erosion often involves sediment shortage, which is usually determined by a decrease of the input from the feeding source (e.g., river basin). River sediment discharge has drastically decreased in the last century, and human activities are most responsible: river damming, river bed quarrying, bank armoring are among the interventions that mostly affected (and still do) bedload and suspended sediment rates [18]. As currents and waves kept on entraining sand from the beaches and distributing it downdrift, no incoming sediments would compensate for the loss, which led to a negative budget and eventually to erosion. Protection structures such as groynes and breakwaters were sometimes built to counteract sand loss: sediments are blocked and accumulate towards the groyne according to the direction of the littoral drift. Indeed the beach experienced accretion updrift, but that was accomplished at the expense of a harsh retreat in the downdrift sector. As sediments cannot be distributed any longer beyond the groyne, the screen effect of hard structures may locally emphasize the erosion impulse already experienced along the coast [19].

Sand redistribution rather than hard protection schemes is now considered the best practice in regard to coastal defense, because it involves managing sediment volumes and does not imply the construction of structures that limit or impede sediment transport [18]. If properly engineered and managed, sand backpassing or bypassing provide significant results in terms of coastal protection and drive to the economy [20,21]. However, these protection schemes cannot be wisely engineered or managed without an insightful understanding of sediment transport patterns and rates. Direction and volumes are required parameters to complete a reasonable assessment of sediment budget for a specific area, which must be a physiographic unit (i.e., a sector of coast that does not have sediment exchange with the adjacent sectors; its sediment budget is usually defined by sediments incoming from rivers or cliff erosion and by sediment loss offshore). All these aspects contribute to increase, and possibly complete, the knowledge about the coastal depositional environment. An in-depth definition of sediment budget would be crucial, but it cannot be reached without a better comprehension of sediment transport.

The challenge for modern coastal managers is to understand the adequate volumes of sediments to replenish a sector of coast, to identify the most suitable site where to perform the beach fill, to evaluate the durability of the intervention in accordance with the estimated rate of sediment loss—all aspects that are tightly linked to sediment management, which is also related to the environmental and socio-economic spheres. Coastal areas have huge naturalistic values, because they are characterized by a variety of different habitats for vegetation and animals, some of which are endangered or at risk of extinction. They are also extremely important from an economic standpoint, as 86 million people live within 10 km from the coastline just in Europe [22]. The whole economy of many regions, even countries, revolves around coastal areas. Commercial activities, supply exploitation, and tourism cannot be sustained without a mindful management of the key resource: sand. Therefore, reliable measurements of sand transport would make the difference between a proper, farsighted planning and a failure.

## 2. State of the Art

Quantification of sediment transport has been addressed in several ways, each showing advantages and drawbacks. In general terms, it is not possible to assume that a specific technique is the best in all situations, because it depends on the reasons why the analysis is being carried out: each method has its best range of application. Three are the most frequently used techniques to measure transport rates: (i) sediment tracing; (ii) volume shift calculation; (iii) use of sediment traps.

Sediment tracing methodologies have long been applied on coastal settings, because they are extremely useful to discern the patterns of particles along or across a beach [23]. The main drawback of tracing experiments is the lack of information in between tracer injection and tracer recovery: initial and final positions are known, not the actual trajectories the particles took to get from point A to point B. First field experiments with natural tracers were done on sandy beaches, analyzing sediment grain-size trends [24,25], sediment composition [26,27], isotopic ratios [28,29], and bioclasts [30,31]. Artificial radioactive [32] and fluorescent [33,34] tracers were also used. Aside from the high percentage of loss, the unresolved issue of these experiments was represented by the fact that the results, even though reliable, were just based on statistics. In the last decade studies on gravel beaches helped minimizing this issue thanks to modern techniques, such as the radio frequency identification (RFID), that allowed to mark individual samples and make them unambiguously identifiable by a code [35,36,37]. One of the most important achievement of this technique has been the exact quantification of the abrasion rate on a coarse-grained beach [38]. Prior to these studies, experiments were carried out using paint [39,40], different lithologies [41], electronic devices [42]: tracer successful recovery did not match that reached using the RFID technology.

As sediment transport is still not measurable during high-energy events, even using modern equipments, assessments about particle movement are usually made based on indirect observations. Topographic surveying carried out before and after storms [43,44,45] is a reliable investigation capable to point out sediment volume shifts in definite time intervals. If performed with accurate instruments—such as DGPS-RTK, laser scanner, multi-beam, etc.—it can provide decent representations of the morphology of a site, enabling comparisons between the pre- and post-storm configurations and the consequent identification of accretion and erosion areas. However, these surveys cannot be carried out on very large sectors, because they are time-consuming. They are most suitable for specific sites, where they can be easily repeated at distinct intervals. Remote sensing also is a technique that is frequently used to address volume shifts [46] through either video-based system image analysis [47,48] and satellite photogrammetry [49,50]. Remote sensing is particularly useful when extended areas have to be investigated: satellites can cover large sectors of the coast but, as they cannot cross clouds, this technique can only be applied during fair-weather periods. Video-based systems do work in bad weather and during storms, but they cover just a few kilometers of the beach where they are installed.

Sediment traps have been used since the last decades of the XX century [51,52], because they provided a direct measurement of the transport rate within specific time frames and flow conditions. As the basic concept is relatively intuitive, traps have been widely applied in many different environments, even though misuse has been reported in literature [53]. By comparison with the other methodologies, traps provide an actual evaluation of transport rates, because sediments can be directly measured as they are collected inside the device. The particles may be analyzed in situ, emptying the trap, and setting it up again for a new time frame of measurements; otherwise, the collected sediments may be brought to a laboratory for further investigations. The more the operator gets to the installation site the more the sampling frequency. In this sense, data collection do provide insights about how much sediment has been transported in a given time frame, but it does not provide any clue about the specific intervals in which transport may have been higher or lower, for instance in accordance with peculiar wind speed and direction. A system that measures the requested parameters in short timespans (minutes), stores and transmits the collected data in real-time would greatly enhance the understanding of sediment transport processes. These datasets may be directly compared to the corresponding wind parameters, providing a wider definition of the relations between wind flow and actual particle movement for specific sites.

Even if Wireless Sensor Networks (WSN) have been widely employed for environmental monitoring [54,55,56], very few solutions exist dealing with the monitoring of coastal areas [57,58]. Moreover, these solutions focus on water quality control [59,60], environmental parameters like air and water temperature [61,62] or oceanographic parameters in general [63]. With the increasing use of WSNs for monitoring marine and coastal parameters it has become of crucial importance to face security issues for transmitted data in order to avoid malicious attacks. In maritime scenarios several practices have been adopted over time to develop protection systems for data traffic produced by WSNs [64,65]. Since many maritime WSNs are based on a not fixed network topology, causing unstable links between nodes, it has also become extremely important the use of Topology Control algorithms for node Mobility [66] and adaptive algorithms which update positions of nodes according to changes in the environment [67]. The question of data security has not been addressed in this paper since the proposed network is expected to be deployed in remote areas where the risk of malicious attacks is virtually inexistent: anyway, this point could be addressed if expecting to deploy the network architecture in anthropized areas.

The solution proposed in this paper foresees the realization of a novel typology of sediment trap, that is able to collect the sand transported by the wind automatically measuring its value and transmitting it in real-time to a remote data acquisition system. The proposed sand trap is then composed by a mechanical structure allowing the sand collection, and by a sensing solution able to measure the amount of collected sand and to transmit the value. Subsequently, this value is correlated with the values of wind speed and direction collected at different levels by a multilayer anemometer-anemoscope structure. Other sensors dedicated to sand transport measurements have been conceived and successfully tested during the last decade. Those systems relied on acoustic [68] and piezoelectric [69,70] sensors, which provided interesting results both in laboratory and in the field. The main drawback of the acoustic system is that it does not record quantitative measurements of sand transport, rather providing data that can be used to make predictions about sand transport. Likewise, the piezoelectric sensors tested by Udo [69] for an extended timespan did not supply quantitative measurements about sediment transport, though the impressive amount of collected data made up for reasonable estimations of sand transport rate. Conversely, Raygosa-Barahona et al. [70] arranged a system characterized by piezoelectric sensors able to record direct measurements of wind-transported sand. Even though the need for calibration of the sensors still remains, the methodology produced significant results in terms of matching recorded wind speed and trapped sand particles. However, the improvements envisioned by the system proposed in the present paper also concern the elevation of the anemometer and the shape of the trap: (i) the anemometers are placed at different elevations (120 cm, 200 cm, 280 cm) because wind speed changes with distance from the ground, especially when wind intensity is low, which may mislead the interpretation; (ii) as described in Section 3, the structure is able to rotate and orient towards wind direction, and not just buried into the sand. As fine sand might overcome the entrance of the trap especially during strong winds, this solution allows to collect all sediment fractions coarser than 63μm, increasing the accuracy of sand transport measurements. Moreover, all these solutions are not provided with remote data transmission capabilities and no real-time data collection is allowed: even if some Wireless Sensor Network architectures for the study of marine sediment transport have been presented [71,72], at our knowledge the proposed solution is the only existing to automatically collect in real-time direct data about aeolian sand transport.

## 3. The Mechanical Structure

The mechanical structure allows the collection of the sand transported by the wind. This device is basically a plastic cylinder with an opening on its side that allows the sand to flow inside it and fall in its bottom where the collected quantity is measured. The structure is conceived to orient to the wind direction through a weather vane and it is provided with two rectangular boxes on its top that hold the electronic circuitry and the transmission module, acting at the same time as ballasts to ease the orientation of the structure.

**Figure 1:**
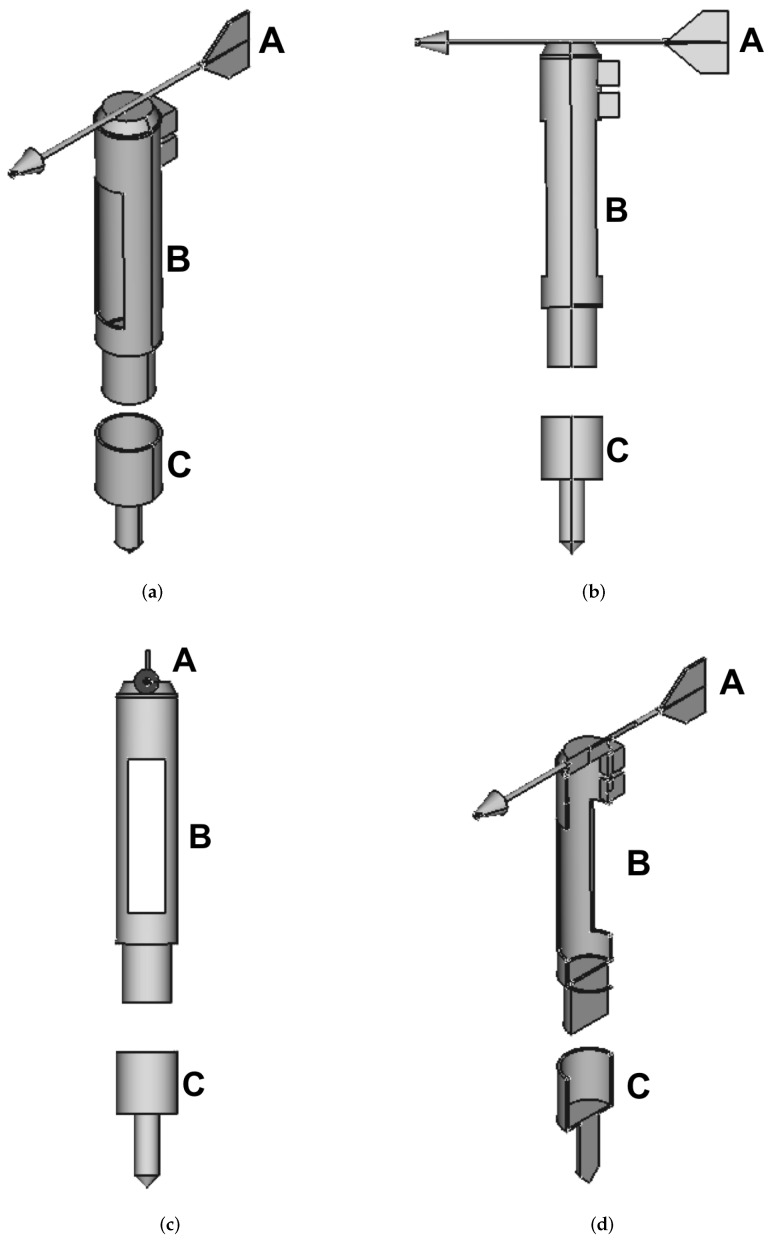
3D model of the mechanical structure: (**a**) axonometric view, (**b**) lateral view, (**c**) frontal view and (**d**) axonometric section. Starting from the top of each subfigure: the weather vane (A), the rotating part with the front opening to let the sand enter (B), the fixed base that will be placed under the beach level (C).

**Figure 2:**
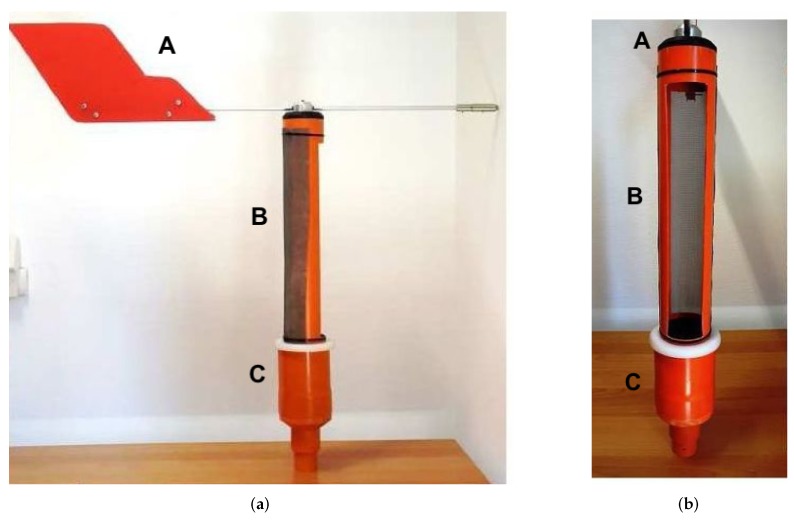
Prototype of the mechanical structure: (**a**) lateral view and (**b**) frontal view. Starting from the top of each subfigure: the weather vane (A), the rotating part with the front opening to let the sand enter (B), the fixed base that will be placed under the beach level (C).

A 3D model of the structure is shown in Figure 1, where four different views of the device are provided: axonometric Figure 1a, lateral Figure 1b, frontal Figure 1c and axonometric section Figure 1d. The developed prototype is shown in Figure 2, where two different views can be seen, lateral Figure 2a and frontal Figure 2b.

The structure is basically divided in two separate parts: the rotating section and the base.

The rotating structure is composed by a 10cm diameter, 96cm high PVC tube with two 7cm×47cm rectangular openings on two sides, cut approximately 20cm above the base. While the front opening is left uncovered, the second opening is closed with a polyester veil with a 63μm mesh, which blocks sand granules but allows wind and finer particles (silt and clay) to flow. Below the lower part of the opening, the joint between the rotating section and the base is realized through an ABS plastic ring, with internal diameter equal to the one of the rotating part and external diameter equal to 14cm: this improvement prevents the sand to filter between the rotating structure and the base, causing possible frictions that might obstacle the rotation. A weather vane, realized connecting a 100cm long metal rod to a metal cylinder on the front side and to a plastic sheet on the back side, is positioned on the top of the PVC tube. The top section of the tube also hosts on its back, fixed with cable ties, two 95mm×65mm×60mm ABS boxes that house respectively the data acquisition and transmission module and the batteries. Two 3D printed bases allow the rectangular boxes to fit with the curved surface of the tube.

The base allows to place the structure in the sand, letting the rotating part free to move according to the wind direction. It is also composed of an ABS tube that hosts the lower section of the rotating structure: this tube is connected to a smaller ABS plastic cylinder that allows the whole structure to be planted deep in the sand avoiding its collapse. Its diameter is slightly larger than the one of the rotating structure (around 105mm): this allows this section to host the lower part of the rotating section letting it free to move. When positioned on the beach, the base is almost totally positioned underground: the sand level is expected to be 1–2 cm lower than the top of the base. The whole structure has been conceived to keep the front opening of the rotating structure as close as possible to the dune or beach surface since wind transported sand usually flows close to the beach surface: this allows to maximize the amount of collected sand.

## 4. The Sensing Structure

**Figure 3:**
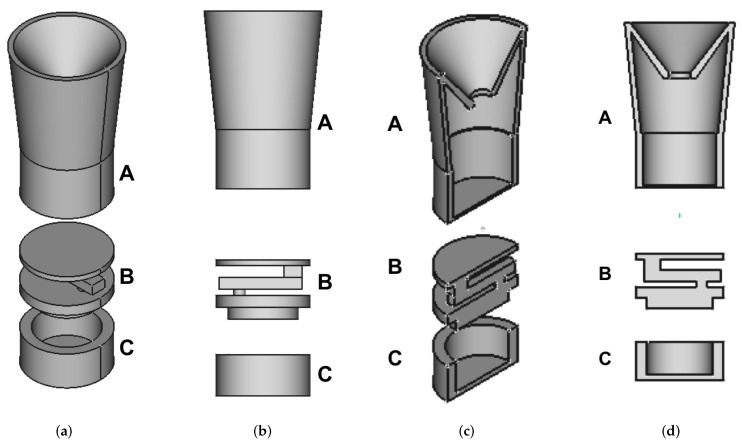
3D model of the sensing structure: (**a**) axonometric view, (**b**) lateral view, (**c**) axonometric section and (**d**) lateral section. The basket used to collect the sand (A) has a flared shape in order to limit contacts with the inner walls of the rotating section that could alter the measured weight; the upper surface is shaped has a funnel. The load cell (B) is bolted to the closing cap of the amplifier housing and it is glued to an upper plate used to hold on the basket. The amplifier housing (C) contains the circuitry and it is closed in order to avoid sand infiltration inside.

In order to measure the amount of sand collected by the trap, a structure integrating both a mechanical part (a basket for the sand) and an electronic part (a load cell coupled with an instrumentation amplifier) has been developed. This device has been designed to be positioned on the bottom of the rotating section of the mechanical structure, perfectly fitting within it and then almost invisible from the outside, thus protected by the atmospherical agents. The 3D model of the structure is shown in Figure 3, with four different views: axonometric Figure 3a, lateral Figure 3b, axonometric section Figure 3c and lateral section Figure 3d. In particular, looking at Figure 3b it is possible to notice the three sections composing the sensing structure: the basket, the load cell section and the amplifier housing.

The basket is basically a 15cm high 3D printed cylindrical container flaring in its upper section to perfectly fit within the bottom of the rotating part of the mechanical structure: this shape has been chosen to reduce as much as possible the impact of possible frictions between the basket and the inner walls of the plastic tube of the rotating section, that could alter the values measured by the load cell. The upper surface of the basket is not open but has been shaped as a funnel in order to direct the collected sand toward the central point of the basket bottom, thus optimizing its distribution inside the basket and then the measure of its weight by the load cell. Once the basket is fully filled with sand, it is expected to be manually emptied. This procedure is made by removing the closing cap of the rotating structure (connected rigidly to the weather vane) and then moving the basket along the inner volume of the rotating structure until the top. This procedure does not require to remove mechanical and sensing structures from their positions and therefore does not interrupt data collection and processing flows; obviously the weight will be erroneous if measured when the basket is not positioned over the load cell. The basket is sized according to specifications given by geologists so it is assumed that during experimental campaigns of about two weeks and excluding exceptional weather conditions, there will be no need to empty the sand collector too frequently. In any case the filling level of the basket can be checked in real time. The realization of an automatic emptying system was discarded due to the fact the basket is positioned below the beach surface and this procedure would have required the realization of a power hungry mechanical solution. Moreover, aside from the technical constraints, the automatic dump of the trapped sediments was not envisaged because a series of laboratory analyses are necessary to round out data collection on the transported sand, such as the average grain-size actually trapped in specific timespans under measured wind and humidity conditions.

The basket is directly positioned on the second component of the sensing structure, i.e., the load cell section. This structure is composed of a lower load cell support, the load cell itself and an upper disk where the load is distributed, as in a scale plate. The lower structure is a 3D printed ABS plastic disc, 10mm high and with 95mm diameter, that acts both as a closing cap for the underlying amplifier housing and as a support for the load cell itself, that is bolted down to it. This structure has a 3mm circular hole used to run the wires of the load cell in order to connect them to the amplifier circuitry. A commercial load cell with a measure range below 5kg has been employed: indeed, the maximum measurable weight is 1.2kg since the basket weighs ∼400 g and the maximum amount of sand collectable by the basket weighs around 800g. The load cell is mounted by fixing one end to the lower support and applying force to the other end: for this purpose it is glued to a second 3D printed, 90mm diameter ABS plastic disc, that holds on the basket where the sand is collected.

The last component of the sensing structure is the amplifier housing: this is a 3D printed, 95mm diameter and 30mm high ABS plastic cylinder. This housing has a cylindrical empty section that perfectly fits the amplifier circuitry: the choice of putting the instrumentation amplifier close to the load cell comes from the need to keep as short as possible the wires linking the cell to the amplifier in order to keep the possible noise as low as possible and then to receive a cleaner signal.

**Figure 4:**
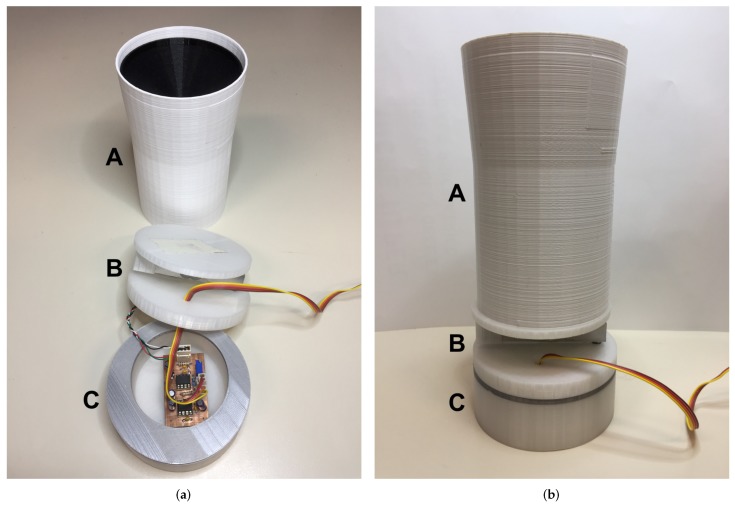
Prototype of the sensing structure: (**a**) decomposed into the individual parts (the basket (A), the load cell fixed to lower support and upper plate (B), the amplifier housing with circuit in the foreground (C)) and (**b**) mounted (the 4-line bus coming out from the amplifier housing connects amplifier and XBee radio module, placed at the top of the rotating part).

The final prototype of the sensing structure can be seen in Figure 4 where it can be seen decomposed into the three sections Figure 4a and mounted Figure 4b.

The load cell provides a differential output, so an INA25P instrumentation amplifier has been chosen to amplify the cell signal: this amplifier allows to set up the gain with a single resistor. Since the load cell output is proportional to 1±0.15 mV for each V of the power supply, powering the cell with a 2.5V voltage, the maximum output voltage will be ∼2.5 mV: this value obviously needs to be amplified.

The amplified output signal will be sent to the analog input of an XBee radio module, provided with a 10 bit ADC (thus giving digital outputs in the 0–1023 range); nevertheless, the reference voltage for the conversion uses a 0–1.2 V range. This means that measured voltages greater than 1.2V will be all converted into a digital value equal to 1023, regardless of their initial values. Hence the amplifier gain must be such as to keep the maximum output voltage of the amplifier, corresponding to the maximum measurable weight, lower than 1.2V. Moreover, the employed load cell has an intrinsic few mV offset that adds up to the effective measurement and gets amplified; for this reason also this amplified offset conditions the choice of the gain resistor of the amplifier. In deciding the gain resistor it is necessary to find a trade-off between signal amplification and level of precision: when the cell is fully loaded the amplified output voltage must stay under 1.2V and at the same time the variations allowed in the weight measurement must be of the order of ±10g. The quantitative computations are detailed below in this section.

The INA25P has a 5V power supply, while the load cell is connected to the reference voltage available from the amplifier, i.e., 2.5V. The output voltage of the amplifier can be calculated according to the following formula:

(1) $$V_{0}=\left(V_{in}^{+}-V_{in}^{-}\right)\cdot G$$

and, according to the INA25P datasheet [73], the gain can be calculated with the following formula:

(2) $$G=4+(\frac{60\;k\Omega }{R_{G}})$$

where $R_{G}$ is the Gain resistance. Assuming that the weight range to be measured is from 400g (the weight of the basket) to 1200g (the approximate weight of the basket filled with sand), it is possible to choose the gain to achieve a ∼1.2 V output with a 1200g weight.

When fully loaded, i.e., with a 5kg weight, the output voltage of the load cell is $V_{out}=2.5$ mV: while its output is linear, with a 1200g load the output voltage will be $V_{out}≃0.6$ mV. Nevertheless, as already stated, when empty, the bridge is not perfectly balanced, but it has a $V_{offset}≃0.2$ mV voltage offset: this means that for a 1200g weight the actual load cell output voltage is $V_{tot}≃0.8$ mV. In order to keep the amplified output voltage below the XBee input voltage limit, a gain resistance $R_{G}=47\;\Omega$ has been chosen, providing a gain

(3) $$G=4+(\frac{60\;k\Omega }{47\;\Omega })≃1280$$

that leads to a total amplified output voltage

(4) $$V_{0}=\left(V_{out}+V_{offset}\right)\cdot G=\left(0.6+0.2\right)\cdot 1280≃1024\;\mathrm{mV}.$$

With the amplification gain equal to 1280, a 100g weight variation corresponds to variation in the output voltage

(5) $$\Delta V_{0}=\frac{0.6\cdot 1280}{1200\;g}\cdot 100\;g=64\;\mathrm{mV}$$

while a 10g variation corresponds to 6.4 mV. Considering that the XBee is provided with a 10 bit ADC, with corresponding $2^{10}=1024$ voltage intervals, its voltage resolution is

(6) $$\frac{1.2\;V}{1024}=1.172\;\mathrm{mV}$$

which coincides with the Least Significant Bit (LSB) voltage, that is the minimum change in voltage that guarantees a change in the output.

This means that every 10g there is a variation of 5.46 voltage intervals. The amplified output voltage can have small variations, in the order of some mV, due mainly to possible inclinations of the mechanical structure. When the sensing structure is tested in the field it might be placed not perfectly orthogonal to the sand level of the beach: because of this slight tilt the sand collected is no more uniformly distributed inside the basket, causing a measured weight a bit lower than the real weight. The maximum admitted slope angle must be measured when the cell is fully loaded and must respect the precision constraint of ±10g on the amplified output voltage. The maximum value of this angle is $\alpha ≃6^{\circ }$ in fact, when the measured weight is 1175g and $\alpha =6^{\circ }$, the actual weight is 1175·cos(α)≃1168g, that satisfies the constraint. When the sand trap is placed in the beach the underground part is sufficiently high to guarantee stability in presence of strong winds; moreover, thanks to the weather vane, the structure is oriented according to wind direction, offering the smallest possible surface to wind. For all these reasons the inclinations actually measured are widely lower than the maximum slope angle. Hence it is possible to conclude that the overall sensing structure is able to measure the sand weight with an approximate precision of ±10g: this degree of precision has been assumed as sufficient by the geologists to assess the evolutionary trends of beaches and dunes.

Once determined the amplification gain, the sensing structure has been calibrated acquiring the amplified voltage output for different sand weights: the values can be seen in Table 1. The lower concentration of measured weights between 0g and 400g is due to the fact that in practice the cell is always loaded with the empty basket, which weighs 400 g. When computing the effective sand weight, this amount is subtracted through software. From 400g on, the required accuracy is greater, so sand weights are taken each 25g. To have a good calibration of the cell also for lower weights, five measurements between 0g and 400g are taken.

**Figure 5:**
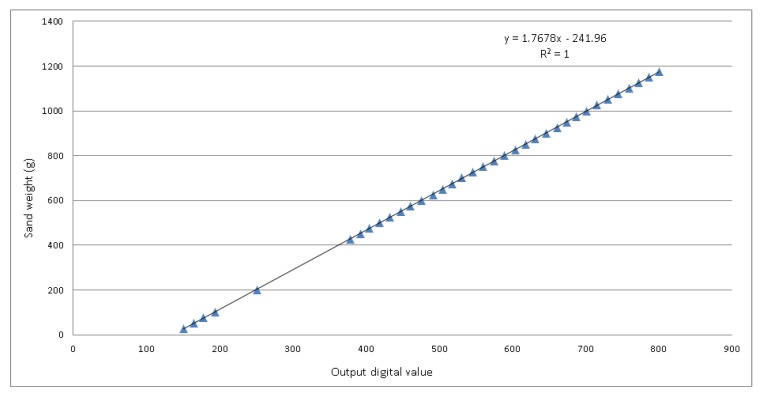
Linear regression of the acquired values. $R^{2}=1$ means that the regressors predict accurately the value of the sand weight using the output digital value.

These values have been used to retrieve their linear regression, determining the function y=mx+q, with m=1.7678 and q=−241.96, that allows to calculate the measured weight from the digital output value. This function has a coefficient of determination $R^{2}=1$: this means that the regressors predict accurately the value of the dependent variable. The graph of the linear regression can be seen in Figure 5.

## 5. Node and Network Architecture

### 5.1. Overall Sensor Node Architecture

The mechanical and sensing structures, together with the data acquisition and transmission circuitry, compose the overall sensor node structure. As already described, the sensing structure is positioned in the bottom of the rotating structure of the mechanical part. Nevertheless, all the electronic components, except for the signal amplifier which is positioned in the bottom of the sensing structure, are put inside the two ABS boxes fixed on the back of the upper part of the rotating structure. A 4-line bus connects the signal amplifier to the two boxes: two lines are devoted to the power line ($V_{CC}$ and Ground) while the other two bring the sensor signal and ground from the amplifier to the transmission module. Since the amplifier and the transmission module are placed respectively in the bottom and in the top of the rotating part, they rotate solidly and there is no risk of winding of the bus around the body of the trap.

The two ABS boxes are devoted to the storage respectively of the batteries and of the electronic circuit receiving the signal from the sensor and forwarding it to the transmission module: the batteries are positioned in the lower one and the transmission circuit in the upper one. Since a minimum 5V voltage is required to power the amplifier and the load cell, 4 1.5V AA lithium batteries put in series have been employed, for a total 6V supply voltage. The choice of two separate boxes is justified from the fact that a separate box for the batteries would prevent the circuitry box from being opened at every battery change, allowing then its possible sealing and a better protection of the most sensible part of the system against atmospherical agents.

**Figure 6:**
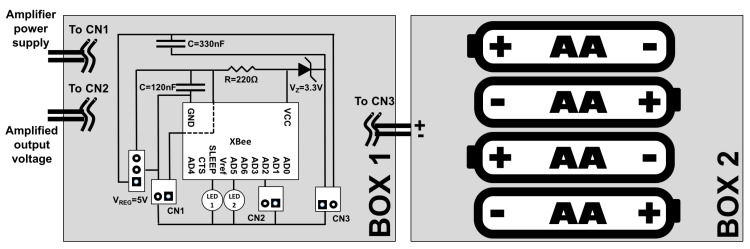
Scheme of the two boxes with the electric diagram of the data acquisition and transmission module. The 2-line connectors CN1 and CN2 are used respectively to feed the amplifier, located in the bottom of the rotating part, and to bring the amplified output voltage in the AD2 input of the XBee. The 2-line connector CN3 is used to bring the supply voltage from the batteries.

Figure 6 shows the 2-boxes architecture and scheme. The 4-line bus is connected to the data transmission module, which is positioned in box 1 (the lower one since it is provided with a hole in its bottom for the bus connection). The transmission module circuitry is very simple, including the XBee module, three 2-line connectors (one to connect the batteries and 2 for the 4 lines coming from the amplifier, one for power and one for signal), 2 LEDs that allow to check whether the module is sleeping or not and when it is transmitting, and additional circuitry for voltage regulation, since the batteries provide a 6V voltage while the amplifier and the load cell must be powered with 5V and the XBee with 3.3V.

**Figure 7:**
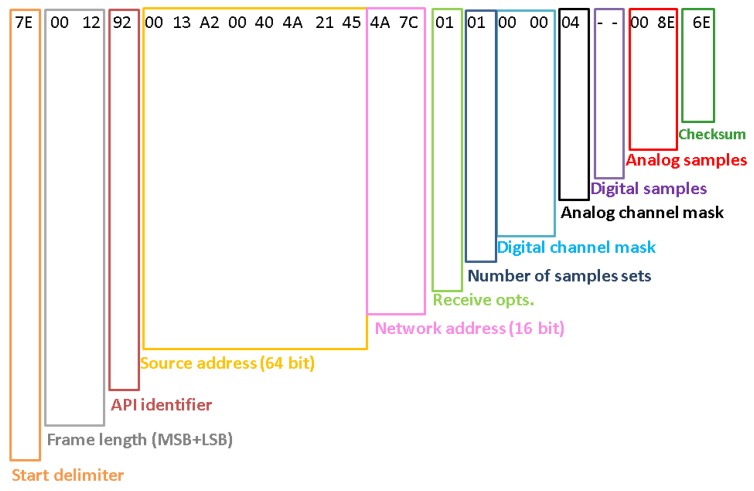
Structure of the automatic sampling data packet transmitted by the node. The sensed value is the Analog Sample which is expressed in hexadecimal values.

**Figure 8:**
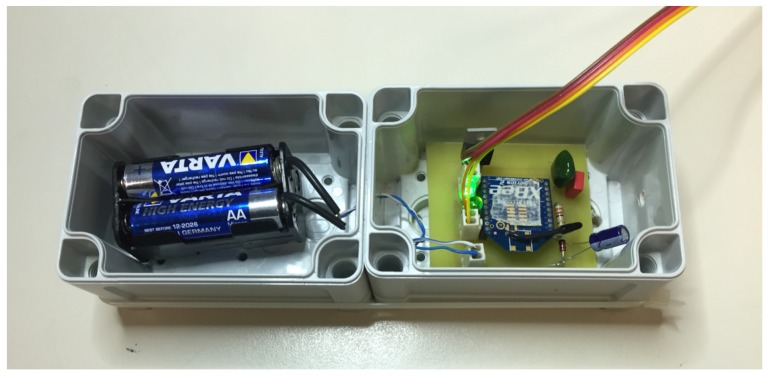
Prototype of the two boxes: batteries are in the left box and circuitry and RF module are in the right one.

The XBee [74] module is used with the automatic sampling option, so periodically it samples its I/O lines in order to send data to the gateway; in this case just the analog input pin connected to the amplified output voltage is enabled. To perform automatic sampling the module must operate in API mode: sampling data are encapsulated in a ZigBee packet with identifier 92 and with a fixed structure shown in Figure 7; in our case payload length is 22 bytes. The maximum XBee data rate is 250 kbps while Serial Interface data rate ranges from 1200 bps to 230,400 bps: in the proposed system it was set at 9600 bps. The ZigBee protocol establishes a bidirectional link and it supports retries and Acknowledgments packets: the recipient sends back to the sender an Acknowledgment when the transmission has been successfully received; if the ACK is not received data are transmitted again up to 2 times. The ideal communication range of XBee modules is around 120 m: this value is obviously conditioned by the environmental features of the deployment scenario. Anyway, in the proposed network all the nodes are in line-of-sight and their distance is lower than 20 m. Moreover, the system is designed to work in non-anthropized and open environments, so it is possible to exclude the presence of surfaces, objects and obstacles responsible of reflecting RF energy. This means that the communication between the nodes and the gateway is ensured. The final prototype of the two boxes can be seen in Figure 8.

### 5.2. Wireless Sensor Network Architecture

The proposed sensor node is expected to be integrated in a larger network architecture including more sand traps and other different typologies of sensors [75], including sand level sensors and structures devoted to the measurement of wind speed and direction. Indeed, this last parameter is fundamental to correlate the amount of transported sand with the wind dynamics at different layers.

Regarding the test infrastructure, three different sensor nodes have been realized:

- The previously described sand trap;
- A multi-layer anemometer-anemoscope structure;
- A gateway node for local data collection and remote transmission.

The multi layer anemometer-anemoscope structure allows the measurement of wind speed and direction at different levels: in particular, the device has been realized to acquire these parameters at a 120cm, 200cm and 280cm height above the beach surface. The structure has been realized using metal poles arranged in a tree structure: three 180cm poles are connected horizontally to a 3 m vertical metal pole. At each of the two ends of the horizontal poles there are placed respectively an anemometer and an anemoscope, for a total number of 3 anemometers and 3 anemoscopes. The 6 sensors are connected to a data acquisition and transmission module which is composed by an Arduino UNO board provided with an XBee shield and an XBee Series 2 radio module: the module is powered by a 10W solar cell connected to a 12V lead-acid rechargeable battery.

The gateway node is composed of an Arduino UNO board connected to a GSM radio shield and to an XBee shield provided with an XBee Series 2 radio module. The gateway acts as a multi-protocol platform gathering all the ZigBee packets coming from the different sensor nodes and forwarding them to a remote data collection centre through GPRS connection. Furthermore, the gateway is in charge of the calculation of the weight by exploiting the linear function obtained in Section 4. At the beginning of the tests, the gateway as well as the anemometer-anemoscope structure, was powered by 10 W solar cell connected to a 12V, 4500mAh, lead-acid rechargeable battery. Nevertheless, this solution has proved not to be adequate to continuously power the gateway during the night hours: indeed, the GSM module is power hungry (the whole gateway has an average 150 mA current absorption when idle and a 500 mA current absorption in transmission with peaks of up to 2 A), to which the current absorption of the anemometer-anemoscope structure has to be added (∼100 mA). The 10W solar cell was characterized by a peak current of ∼600 mA: anyway, this current is supplied only in conditions of perfect sunlight that are available only in sunny days and in peak hours. During the night, the gateway (and the anemometer-anemoscope structure) is powered only through the battery that has an ideal capacity of 4500 mAh: since the overall current absorption is around 250 mA, with peaks up to 2 A, the battery is then expected to power them for a maximum of 14/15 h. During the day, the cell has to simultaneously power the nodes and to recharge the battery and its power supply is not sufficient to perform both the operations. As a consequence, the discharge rate is higher than the charge rate and the solar cell is not sufficient. For this reason, during the continuation of the experimentations, the 10 W solar cell has been replaced with a 60 W one that is characterized by a 3.45 A peak current and that proved to be sufficient to continuously power the gateway and keep the battery charged. A more efficient solution could be set up by accurately measuring the single consumption but fell out of the scopes of this paper.

The overall network architecture is based on a ZigBee-based mesh topology where all the sensor nodes are configured as Zigbee Routers, while the gateway node is configured as the ZigBee Coordinator. The Coordinator is also provided with GSM global connection: this allows it to transfer the measured weight to a cloud infrastructure in charge of storing it and making it available through a web page. The data transfer is performed through an HTTP GET request to a Java Web Application which manages the data reception and its storage in a MySQL database, as well as its visualization.

## 6. Tests and Results

The sensor node and the overall network architectures have been tested both in laboratory and in the field through ad-hoc measurement campaigns. The laboratory tests have allowed the calibration of the sensor, as described in Section 4 as well as preliminary analysis on data transmission timings and node power consumption (and then possible life time).

Regarding the data acquisition and transmission timings, in the test phase a 15 s sample rate has been defined for data collection: this value allows to collect three values for each minute of activity of the XBee radio module because when the XBee wakes up from the Sleep Mode the first few seconds are used for activation, association to the network and polling procedure to determine if the Coordinator has indirect messages for the XBee itself. It will be then possible to average the three values in order to have a more accurate reading: the average will be calculated during the post-processing phase that will be carried out on the data stored in the cloud infrastructure.

One of the main objectives during the design of the sensor nodes was to reduce as much as possible the power consumption of the sensor node and then to ensure a long life time, at least in the order of some weeks. Since the node is expected to be powered with AA lithium batteries, whose capacity is generally measured mAh, also the consumptions of the single components have been quantified in the same way. Laboratory measurements performed by powering the system with a laboratory DC power supplier and then measuring the absorbed current with a digital multimeter showed the following results for battery capacity dissipation:

- XBee module: if not put in sleep on average 40mAh;
- INA125P instrumentation amplifier: ∼3 mAh;
- LD1085V33 Voltage regulator: ∼5 mAh.

The current absorption of all the other components can be considered negligible with respect to these three.

The calculations of the node life time have been performed assuming a battery capacity of 3000mAh, which is a common value for high energy lithium AA batteries. If assuming the XBee mode to be never put on sleep, the total consumption of the sensor node will be:

(7) $$C_{tot}=C_{xbee}+C_{amp}+C_{reg}=40\;\mathrm{mAh}+3\;\mathrm{mAh}+5\;\mathrm{mAh}=48\;\mathrm{mAh}$$

The life time of the node will be then:

(8) $$L=\frac{C_{batt}}{C_{tot}}=\frac{3000\;\mathrm{mAh}}{48\;\mathrm{mAh}}=62.5\;h≃2.6\;\mathrm{days}$$

While this value is very low, it can be notably increased enabling the sleep mode of the XBee: in this case, its consumption drops from 40mAh to few μAh that can be considered negligible. With a $d=\frac{1}{60}$ duty cycle, that means 1 min of activity every hour, the average consumption value for the XBee drops to:

(9) $$C_{duty\_cycle}=\frac{C_{xbee}}{60}=\frac{40\;\mathrm{mAh}}{60}=0.667\;\mathrm{mAh}$$

The overall node consumption drops in turn to

(10) $$C_{tot}=C_{duty\_cycle}+C_{amp}+C_{reg}=0.667\;\mathrm{mAh}+3\;\mathrm{mAh}+5\;\mathrm{mAh}=8.667\;\mathrm{mAh}$$

And the life time grows to

(11) $$L=\frac{C_{batt}}{C_{tot}}=\frac{3000\;\mathrm{mAh}}{8.667\;\mathrm{mAh}}≃345\;h≃14\;\mathrm{days}$$

This value is mainly due to the presence of the Voltage regulator, then it could be notably reduced by introducing more efficient, even if more expensive, voltage step-downs, or even a transistor working as a switch. Adding on board data processing would allow to reduce the number of transmissions and then the time of activity of the XBee: anyway the 14 days life time value is sufficient to carry out a mid term data acquisition campaign. Moreover, if long term monitoring campaigns are expected to be carried out the sand basket needs to be periodically emptied: this means that a manual intervention on the sensor node is periodically mandatorily required; during this phase it is then possible to change the batteries.

**Figure 9:**
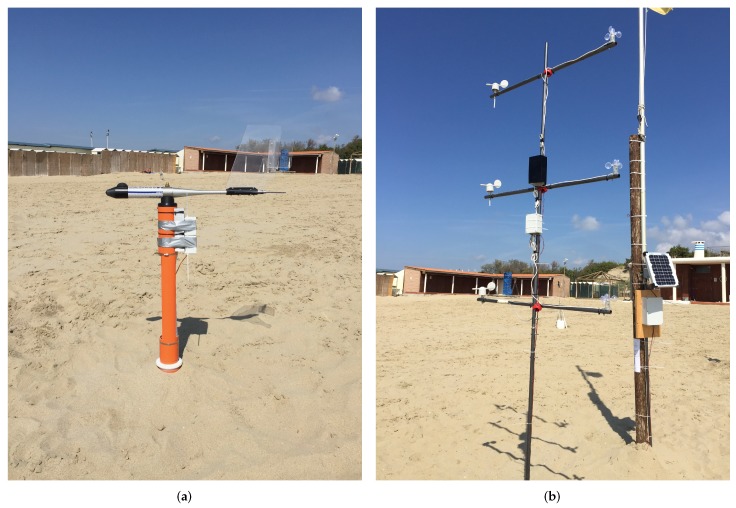
The nodes deployed on the Marina di Tirrenia beach, Pisa, Italy: (**a**) the sand trap and (**b**) the anemometer-anemoscope structure. The upper black box fixed in the middle of the anemometer-anemoscope structure houses the Coordinator. In the background it is possible to notice the 10 W solar cell powering the two nodes.

**Figure 10:**
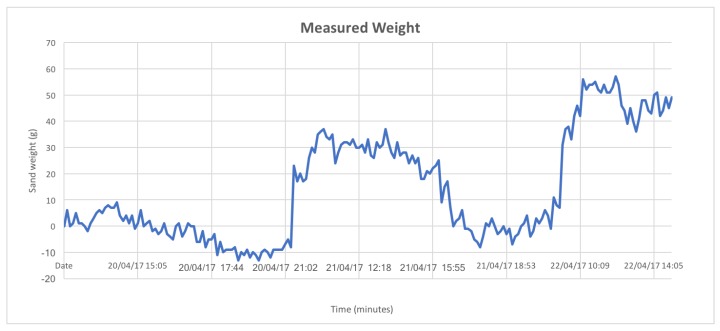
Collected sand weight during the data acquisition campaign carried out on the beach of Marina di Tirrenia.

**Figure 11:**
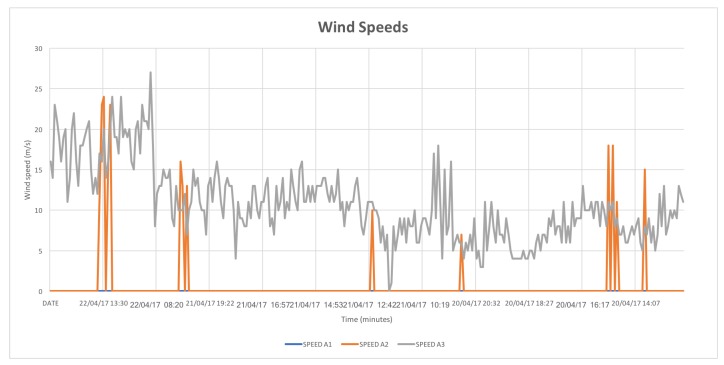
Wind speed measured by the three anemometers during the data acquisition campaign carried out on the beach of Marina di Tirrenia. A1 is the lower anemometer placed at 120 cm height, A2 is the middle anemometer placed at 200 cm and the A3 is the higher one placed at 280 cm.

Following the laboratory test, the sensing infrastructure has been tested in the field with a 48 h data acquisition campaign carried out on the beach of Marina di Tirrenia, Pisa, Italy from 20 April 2017 to 22 April 2017. The deployed devices can be seen in Figure 9. For these tests the sleep period of the sand trap was shortened in order to have a larger number of datasets: in particular, a 20 min sleep period was set up (with one minute of activity). The anemometer-anemoscope was set to transmit a reading each 5 min. The results of the data acquisition can be seen in Figure 10 and Figure 11. Figure 10 shows the trend describing the amount of collected sand by the sand trap, while Figure 11 shows the trends of the wind speeds: it is possible to notice that Speed A1, which corresponds to the wind speed measured by the lowest anemometer, is always 0, while Speed A2, corresponding to the middle anemometer, is almost always 0 except for 6 short periods when it reaches values up to ∼20 m/s. Speed A3, corresponding to the highest anemometer, is always ≠0, with values ranging from ∼5 m/s to ∼20 m/s.

The following considerations have to be made analyzing the chart:

- The basket was emptied around 5:00 p.m. on the second day in order to weigh the collected sand to check the correctness of the measured weight.
- Due to a few peaks, the data show a degree of precision which is slightly worser (±12/13g) than the predicted one (±10g). Nevertheless, this result could be improved through an in field calibration of the load cell that would then hold in consideration the environmental features of the real scenario, i.e., the perfect perpendicularity of the structure, the pressure of the sand on the underground external walls of the base of the trap, the sand humidity, the impact of winds, the temperature, the plastic dilatation due to the sun exposure, etc.;
- Looking at Figure 10 it is possible to notice that the basket remained empty from the beginning of the experiment (around 12:00) to around 21:02 of the same day: during this period the sand weight values are in a +9g\−13g range which is compliant with the required accuracy. The slight negative trend may be due to changing environmental conditions, for example a slight inclination of the trap or the presence of humidity. After 21:02 the measure values grow in a +17g\+37g range: this value has been validated by measuring the weight of the sand with a scale after the emptying of the basket (26g). Two peak values (9g and 15g) were measured during the emptying procedure and, even if reported on the chart, should not be taken into account. Following the emptying, the measured weight remained 0g for about 7 h, going then up to a value in the +33g\+59g range which is compliant with the weight value measured through a scale at the end of the experiment (47g);
- The presence of negative values is due to the calculation of the sand weight carried out by the Coordinator that subtracts to the measured value the offset weight of the basket: this means that when a 0g value for the sand weight is calculated, the actual measured value by the cell is 400g (the basket weight). Indeed, due to the ±10g precision degree, when the basket is empty the minimum acceptable value for the sand weight can be ∼−10 g;
- The amount of collected sand is vary small. This suggests that the most part of aeolian sand transport is due to superficial winds that during the experimentation where almost absent;
- A few data sets were lost, mainly due to the low GPRS coverage on the beach that led sometimes the GPRS gateway to disconnect from the network and then to reconnect again after a short span of time.

## 7. Conclusions

The aim of this paper was to present a novel solution for the analysis of aeolian sand transport on sandy beaches and dunes. The proposed solution has proved to be an efficient tool to collect direct measurements of this parameter, which was usually assessed with a statistical approach so far. As a matter of fact, no such technological solutions had ever been developed at all. Moreover, the system is able to transmit the measured values through Internet connection: this allows the data to be available remotely and in real time. The availability of this kind of data is crucial to better define the coastal dynamics on beach affected by the coastal erosion phenomenon because it provides actual quantification of how much sediment can be transported under specific conditions of wind. Once the system will be operating for longer timespans, it would be also possibile to match transport data with air and ground humidity, which is a parameter that greatly affects the chance of sand granules to move.

The described system has been tested in the field, proving its functioning, and is expected to be widened to include more sensor nodes and then to be employed for in field measurement campaigns in the next future. Even if the tested infrastructure has proved to satisfy the requirements defined by the geologists in terms of accuracy and life time, further work could be carried out to improve the degree of precision, first of all through the design of a more efficient amplification circuit and then identifying a more accurate load cell. Similarly, additional work could be carried out to further optimize the node consumption by adding more energy efficient components like ultralow quiescent current low-dropout linear regulators or duty-cycling procedures to turn off not used sections of the system.

In conclusion, the proposed system may represent a useful tool for the study of the dynamics not only in coastal areas but also in all the environments characterized by the presence of sand such as for example the desert areas.

## Figures and Tables

**Table 1:** Measured data for the calibration of the load cell.

Weight	Output Digital Value	Sand Weight	Output Digital Value
25 g	151	750 g	560
50 g	165	775 g	575
75 g	178	800 g	589
100 g	194	825 g	605
200 g	251	850 g	618
425 g	379	875 g	632
450 g	393	900 g	647
475 g	405	925 g	662
500 g	419	950 g	675
525 g	433	975 g	688
550 g	448	1000 g	702
575 g	461	1025 g	716
600 g	476	1050 g	731
625 g	492	1075 g	745
650 g	505	1100 g	760
675 g	518	1125 g	773
700 g	531	1150 g	787
725 g	546	1175 g	801
